# Miniaturized shared aperture multiband antenna for wireless biomedical applications

**DOI:** 10.1371/journal.pone.0349676

**Published:** 2026-06-04

**Authors:** Abdul Rehman Chishti, Abdul Aziz, Muhammad Nawaz Abbasi, Khaled A. Aljaloud, Ali H. Alqahtani, Rifaqat Hussain

**Affiliations:** 1 Department of Information and Communication Engineering, Faculty of Engineering, The Islamia University of Bahawalpur, Bahawalpur,‌‌ Pakistan; 2 College of Engineering, Muzahimiyah Branch, King Saud University, Riyadh, Saudi Arabia; 3 Antenna and Electromagnetics Research Group, School of Electronic Engineering and Computer Science, Queen Mary University of London, ‌‌London, United Kingdom; University of Leicester, UNITED KINGDOM OF GREAT BRITAIN AND NORTHERN IRELAND

## Abstract

This study presents a compact, folded dipole multiband shared-aperture antenna that operates effectively across both sub-1 and sub-6 GHz frequency bands using a single radiating structure—constructed on an FR-4 substrate. Design measures $16.40\times 10.50\times {1.52mm}^{3}$ (0.0386λ×0.0247λ×0.0036λ), (0.431λ×0.276λ×0.040λ), (0.512λ×0.328λ×0.048λ), resonates at the bands of 0.431-0.435 GHz, 4.75-4.96 GHz, and 5.64-5.88 GHz, thus having a flexibility to a wide range of applications. The proposed antenna is a folded dipole built on the upper layer, and the coaxial feeds run to horizontally and vertically oriented strips on the backside layer. This arrangement enables simultaneous use along with various frequency bands in a single physical structure by taking advantage of shared-aperture concept thereby achieving space economy and operational efficiency. By applying a miniaturization plan based on lumped element integration, the radiating system guarantees a reduction of 80% in the dimensionality, and remains functionally intact. Besides, the integrated measurement subsystem incorporates a cancer-related analyte which mimics the electromagnetic nature of cancerous cellular organisms and thus a detectable spectral change at 5.7 GHz. The implication of this phenomenon is the initiation of an oncogenic presence diagnostic indicator. The concomitant capability of operating in multiple bands and smaller footprint make the antenna especially favourable to use in the context of operating in healthcare infrastructures for biomedical platforms along with wireless communication applications.

## Introduction

Antennas are an important part of modern world of wireless communication [1–5], especially 5G communication. Working in specific frequencies, they maintain constant connections of the devices in different settings, at home, at workplace, or on the move. Recent advancements like MIMO and beamforming improve signal strength and reliability even more, particularly in cities with a large population density with numerous devices competing to get the communication resources.

The recent developments in antenna design have greatly expanded its use, extending well beyond the old-fashioned communication roles and making notable discoveries in the field of biomedical uses [6,7]. Theoretical uses includes automotive systems with antenna-based systems upon them [8], satellite platforms and other systems with antennas on them [9–11], biomedical environments and aircraft aboard them respectively. Antennas used in the biomedical context not only transmit information, but also facilitate information tracking, surveillance, and other applications, as well as monitoring capabilities [12–14].

Designing a miniature antenna is also essential since it can be fitted easily in small gadgets. The electrically small antennas (ESA) is a result of miniaturization of the antenna. Such a design however requires certain technologies and the corresponding impedance-matching network that is dependent on the application [15]. The inverse relationship between Q factor and antenna bandwidth has a negative impact on ESA as it is limited in bandwidth. Further, the setback is due to matching of impedances, thus nullifying the overall ESA performance.

First, the antennas were placed side by side to induce the behavior of multiband antennas. The novelty in this design is that a common aperture of an electrically small antenna has been implemented, an important breakthrough in small antenna designs. The shared aperture allows shared physical space to be used by various radiating elements or functions, allowing other frequency bands or functionalities to share the aperture. This is done by decreasing the size and complexity of the antenna in general but maintains multiband operation. In the case of electrically small antennas where space is the most important factor, such a technique is the best way to maximize the use of space without reducing performance.

Two or more antennas at different frequency bands are separately fixed into one radiating topology in a shared aperture which subsequently provides a compact form factor, high efficiency, and reduced mass, which has been reported by [16,17].

The combination of two or more antennas in a common aperture faces challenges of the spatial distance between the antennas and the dimensions of the radiating elements. To address these challenges while maintaining a compact design, microstrip antennas are often preferred for their low mass and ease of integration with the feeding network. Most designs of shared aperture antennas have focused on applications suitable for 5G technology [18], particularly in the sub-6 GHz and mm-wave bands.

For biomedical application recommended bands include 433–434MHz, 608–614MHz, 868-868.6MHz, 902.9-928MHz, 1395–1400MHz, 1427–1432MHz and 2.4-2.5GHz [19]. For 5G communication, new bands are added that include sub-6 GHz and millimeter-wave to achieve wireless communication with higher capacity [20,21].

## Related work

Authors in [22] have designed a folded dipole operating at a 2.4 GHz band using multiple lumped elements, where frequency shifting at sub-1 GHz was achieved. Multiple lumped elements bring complexity to the design, and designing such an antenna is tedious. Several authors have worked on the design of ESA. The manuscript [23] presents the design of an electrically small antenna with dimensions of 0.165 × 0.164 × 0.006$\lambda_{3}$, fabricated using Rogers Duroid 5880 substrate at 2.4 GHz. However, no information related to cancer detection using this ESA was discussed. Authors have suggested a method to achieve better (Q Factor) and reasonable bandwidth with antenna dimensions 30 × 30 *mm*^2^ operating at 1.575-GHz in [24]. The proposed ESA gives 2.35% bandwidth while Q = 30. For the design of a shared aperture antenna, most authors have correlated the antenna design suitable for 5G technology operating at sub-6GHz and mm-Wave band. A low-profile triband antenna suitable for 5G massive MIMO application was proposed in [25], having dimensions of 0.69 $\lambda_{L}$ × 0.69 $\lambda_{L}$ × 0.177 $\lambda_{L}$ operating at sub-1 GHz, sub-3 GHz and sub-4 GHz bands. A shared aperture suitable for vehicle-to-vehicle 5.9GHz and 5G communication (28-GHz) was proposed in [26] with dimension (0.65 $\lambda_{o}$ × 0.43 $\lambda_{o}$). Results indicate that bandwidths of 41.6% and 6.66%, with antenna gains of 5.80 and 8.46 dBi, respectively. Authors in [27] have proposed a shared aperture antenna suitable for 5G devices, covering the eight bands from 1 GHz up to 5.6 GHz on a Rogers RO4350 substrate providing a minimum bandwidth of 1 GHz. However, the authors have not discussed any biomedical aspect of this design.

A low-profile triband antenna suitable for 5G massive MIMO application was proposed in [25], having dimensions of 0.69 λ ×0.69 λ × 0.177 λ operating at maximum frequencies of 0.96 GHz, 2.7 GHz, and 3.8 GHz.

Antenna’s dimensions are substantial, making it impractical for integration into compact smart devices. A shared aperture suitable for vehicle-to-vehicle (V2V) communication at 5.9 GHz and 5G communication operating at (28-GHz) was proposed in [26] with dimension (0.65 λ × 0.43 λ) operating as a dual-frequency antenna. Results indicate that bandwidths of 41.6% and 6.66%, while antenna gains of 5.80 dBi and 8.46 dBi, are achieved. However, the concept of a shared aperture in a miniaturized antenna is yet to be explored.

The main contribution of this research includes:

- A Miniaturized antenna is designed to achieve resonance at sub 1 GHz and sub 6 GHz; horizontal and vertical pairs of parallel strips at orthogonal configuration are used at the bottom layer of the substrate to provide excitation.
- A Simplified planar structure has been proposed with reduced lumped elements. Impedance matching at 50Ω was achieved without using any Balun transformer, despite using multiple coaxial feeds.
- The proposed antenna shares the same folded dipole geometry despite its different feeding structures, allowing it to cover the sub-1 GHz (434 MHz) and sub-6 GHz (4.8 GHz and 5.7 GHz) bands, which indicates the distinctive features of this antenna design, respectively.
- The addition of a cancer analyte into the system will cause a frequency shifting, which will enables the prompt detection of malignancies.

### Antenna design details

This antenna is built on a compact FR-4 substrate, measuring $16.40\times 10.50\times 1.52\;{mm}^{3}$, in terms of λ the dimensions are mentioned as: At 0.433 GHz: 0.0386λ×0.0247λ×0.0036λ, At 4.85 GHz: 0.431λ×0.276λ×0.040λ, and at 5.76 GHz: 0.512λ×0.328λ×0.048λ, with a relative permittivity of $\epsilon_{r}=4.3$.

The top layer contains a folded dipole patch, whereas the bottom layer features two pairs of parallel strips added to ensure dual-port feeding. In this configuration, one pair of strips is oriented vertically to feed Port 1, and the other is oriented horizontally to feed Port 2.

**Fig 1:**
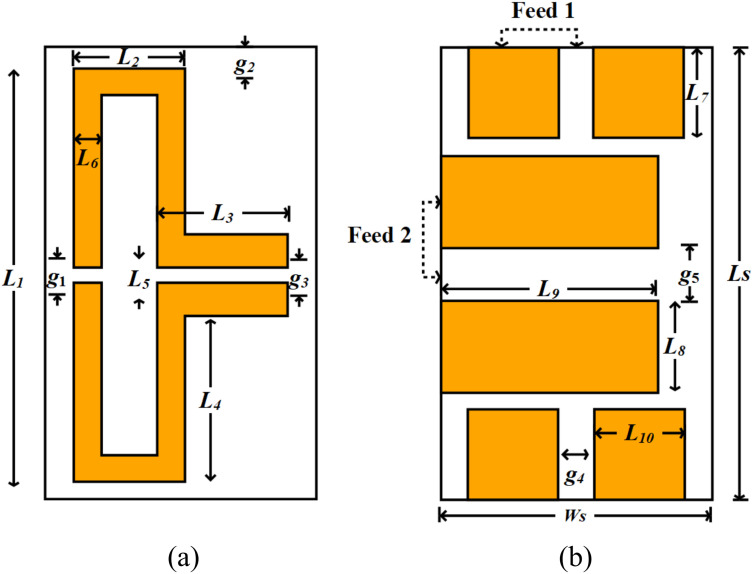
Shared Aperture Folded dipole patch antenna (a) Top View (b) Bottom View.

This design enables multi-band operation suitable for biomedical applications while remaining small and portable. Fig 1 shows the geometry of the top and bottom of this antenna, along with its antenna dimensions. This antenna originally resonates at 2.17 GHz. After applying the miniaturization scheme using a lumped-element (inductive) component, the antenna now resonates at 434 MHz. This indicates that, with the same dimensions, an 80% miniaturization has been achieved in this design.

The proposed antenna is fabricated with compact dimensions of $16.40\times 10.50\times 1.52\;{\text{mm}}^{3}$. In terms of the guided wavelength $\lambda_{g}$ (using $\epsilon_{\text{eff}}\approx \epsilon_{r}$), the electrical sizes at the operating frequencies are:

- 0.433 GHz: $0.0386\lambda_{g}\times 0.0247\lambda_{g}\times 0.0036\lambda_{g}$
- 4.85 GHz: $0.431\lambda_{g}\times 0.276\lambda_{g}\times 0.040\lambda_{g}$
- 5.76 GHz: $0.512\lambda_{g}\times 0.328\lambda_{g}\times 0.048\lambda_{g}$

Compared to a conventional quarter-wavelength antenna at 0.433 GHz (which requires approximately 83.5 mm in length along the substrate), this design achieves a significant reduction of ∼80% in the longest dimension 16 *mm*, while maintaining multiband performance.

FR-4 substrate ($\epsilon_{r}=4.3$) was selected over low-loss alternatives (e.g., Rogers or Teflon) mainly due to its very low cost, excellent mechanical robustness, and widespread commercial availability, which are particularly advantageous for proof-of-concept biomedical sensor prototypes. Moreover, at the targeted sub-1 GHz to 6 GHz frequencies used for dielectric-based cancer cell detection, the moderate dielectric loss of FR-4 remains acceptable and does not significantly degrade the measurable frequency-shift sensitivity, while enabling rapid, affordable fabrication of disposable or low-volume biosensing devices.

To achieve resonance in the sub-1 GHz range, we integrated a lumped-element (inductive) component into the folded dipole patch. The position of this inductor is placed at the midpoint of the outer left path of the folded dipole, i.e., *L*_1_/2 = 7.6 *mm*, while the spacing between the inductor paths is labeled as *g*_1_ = 0.6 *mm*. By altering the value of the inductance value between 100 nH to 200 nH, resonance curve can be shifted to reach around the target frequency. Applying tuning of the antenna’s parameters, the antenna performance is improved, thus result in antenna resonating at sub-1 GHz range 0.431−0.435GHz, along with further multifrequency bands of: 4.75−4.96GHz, and 5.64−5.88GHz.

The antenna’s performance is enhanced by adding a Defective Ground Structure (DGS) to the bottom-most layer of the antenna. The resulting design works best, which is due to its small size which allows to achieve a decreasing in of the overall size, at the same time, enhancing impedance matching. The addition of two ports in each pair of parallel strips also contributes towards the performance of the antenna thus facilitating the multi-band operation that may be applicable to both biomedical and communication applications.

### Parametric analysis

Simulations of the proposed antenna are performed in CST Studio Suite. A parametric sweep is applied to several antenna parameters to optimize the antenna for both biomedical and 5G communication bands. The final optimized dimensions are listed in Table 1.

**Table 1:** Optimized Parameters of the Proposed Folded Dipole Antenna.

Parameter	Value (mm)	Parameter	Value (mm)	Parameter	Value (mm)
*L*_1_	15.20	*L*_2_	3.60	*L*_3_	6.70
*L*_4_	5.60	*L*_5_	1.50	*L*_6_	1.20
*L*_7_	4.40	*L*_8_	2.80	*L*_9_	7.55
*L*_10_	3.80	*L*_*s*_	16.40	*W*_*s*_	10.50
*g*_1_	0.60	*g*_2_	0.60	*g*_3_	1.00
*g*_4_	0.60	*g*_5_	1.20	*h*_*s*_	1.52

### Reflection coefficient curves

Several antenna parameters are changed, including the length of the folded dipole arm (*L*_3_), back strip length (*L*_7_), back strip width (*L*_10_), and the inductance (*L*_*ind*_). Their reflection coefficient curves are obtained. This section shows the results obtained from variations in these parameters. These curves are studied carefully to achieve the required optimization of the proposed shared aperture antenna.

### Variations in arm length *L*_3_

**Fig 2:**
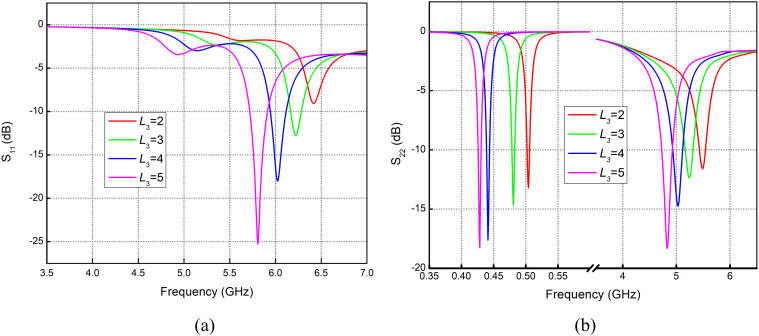
Reflection coefficient for variation in *L*_3_ with feed applied at orientation (a) Vertical pair of strips. (b) Horizontal Pair of Strips.

Change in the arm length of the folded dipole (*L*_3_) causes the increase or decrease in the electrical length of the folded dipole; hence, frequency shifting is noticed. Fig 2(a) represents the reflection coefficient curve due to the feed 1 applied at the top vertical pair of parallel strips. Resonance can be seen at 5.7 to 6.4 GHz with arm length variations from 2 to 5 mm. Applying feed at a horizontal pair of parallel strips results in dual-band resonance at sub-1GHz and sub-6GHz. Fig 2(b) illustrates the curves where resonance at sub 1GHz (0.4 to 0.6 GHz) is achieved, along with 4.7 to 5.6 GHz at higher bands (sub-6 GHz) with varying arm lengths from 2 to 5 mm.

### Variations in Inductance (*L*_*ind*_)

**Fig 3:**
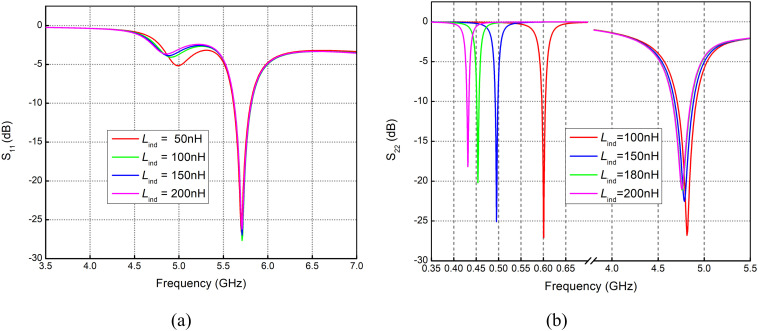
Reflection coefficient for variation in Lind with feed applied at orientation (a) Vertical pair of strips. (b) Horizontal Pair of Strips.

Reflection coefficient curves, as shown in Fig 3, illustrate the variations in inductance from 100 nH to 200 nH. Such variation indicates the least impact at higher frequencies; however, a significant shift in *S*_11_ is noticed at the sub-1 GHz band.

### Variations in back strip length (*L*_9_)

**Fig 4:**
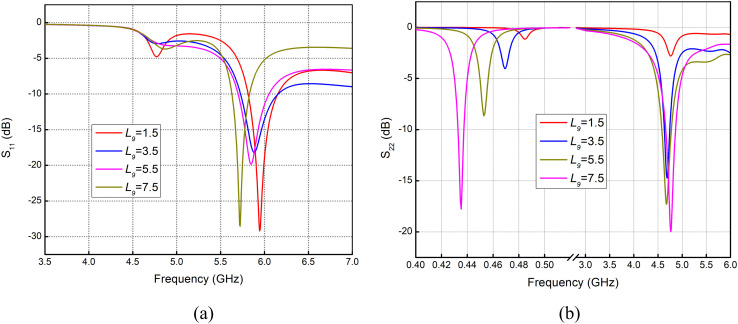
Effect on Reflection Coefficient for variations in *L*_9_ in (mm) (a) *S*_11_ (b) *S*_22_.

Applying a parametric sweep from 1.5 to 7.5 mm to the length *L*_9_ (horizontal pair of parallel strips), a shifting of the resonance curve to a lower frequency, as indicated in Fig 4(a), can be noticed. Fig 4(b) shows the reflection coefficient *S*_22_, where improved impedance matching can be seen at the lower frequency band, while a similar trend can be seen for the 4.7 GHz band. However, only a significant frequency shift is noticed at the sub-1 GHz band.

### Variations in Back strip length (*L*_10_)

**Fig 5:**
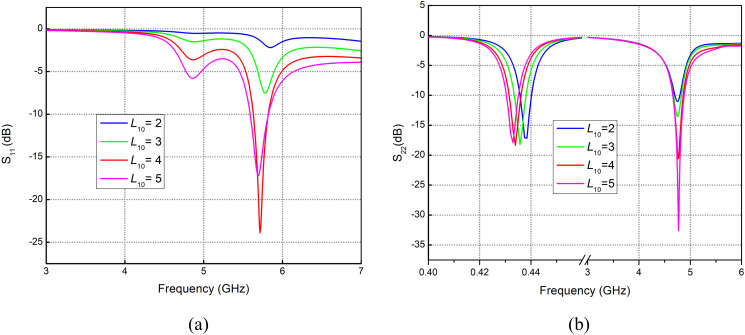
Effect on Reflection Coefficient for variations in *L*_10_ in (mm) (a) *S*_11_ (b) *S*_22_.

*L*_10_ represents the horizontal length of the vertical pair of parallel strips (port 1 feeding). Variation in this parameter results in reflection coefficient curves depicted in Fig 5(a), while Fig 5(b) shows resonance at the sub-1 GHz band, where frequency shift from 0.439 GHz to 0.43 GHz is observed with change in length (*L*_10_). In contrast, improved impedance at sub-6 6GHz is achieved.

**Fig 6:**
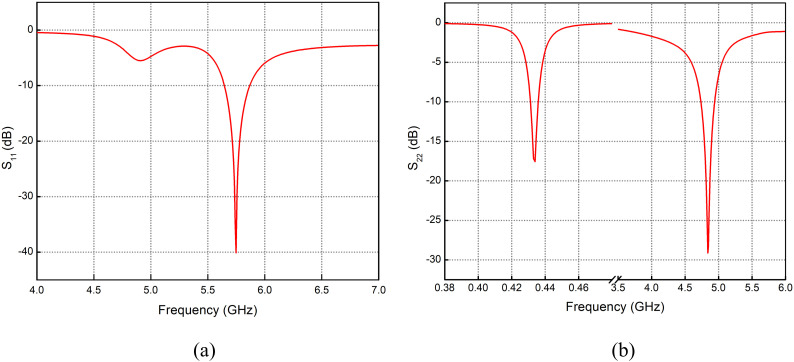
Return Loss with feed applied at (a) Vertical parallel strips (b) Horizontal parallel strips.

The final design of the antenna contains two feeds; one is applied at the horizontal parallel strips producing resonance at 434 MHz and 4.8 GHz, as shown in Fig 6(b), while the feed applied at the vertical parallel strips produces resonance at 5.8 GHz, as shown in Fig 6(a). The bandwidth of 223 MHz has been achieved at 5.74 GHz, 207 MHz at 4.84 GHz and 4.5 MHz at 434 MHz (sub-1 GHz). Realized gain of −31 dBi, −3 dBi and −6.8 dBi at resonance frequencies of 434 MHz, 4.8 GHz and 5.7 GHz, respectively, is achieved.

Variations in horizontal feedline strip *L*_9_ and vertical feedline strip *L*_10_ are shown in Fig 4 and Fig 5. Varying *L*_9_ causes a frequency shift from 5.95 GHz down to 5.6 GHz. At the same time, improved resonance and noticeable frequency shifting are also observed in the sub-1 GHz span. At 4.7 GHz band, resonance improves significantly, with return loss rising from −0.2 dB to around −20 dB. The same effect can be seen in Fig 5(a) and Fig 5(b), where improved return loss at 5.6 GHz with variation in *L*_10_ from 2 to 5 mm is observed, while at 4.7 GHz the return loss improves from −10 dB to −34 dB with variation in *L*_10_ from 2 to 5 mm.

### Ethics statement

This research did not involve any human participants or their data. Therefore, participant consent was not applicable.

### Current distribution

The existing distribution plays a critical role in understanding the effect of different parts of the antenna on its functioning and performance particularly on matters of frequency shift and the impedance matching. When examining areas where the current is at its highest or lowest, it is possible to identify the places that require adjustment to enhance the overall operation of the antenna.

**Fig 7:**
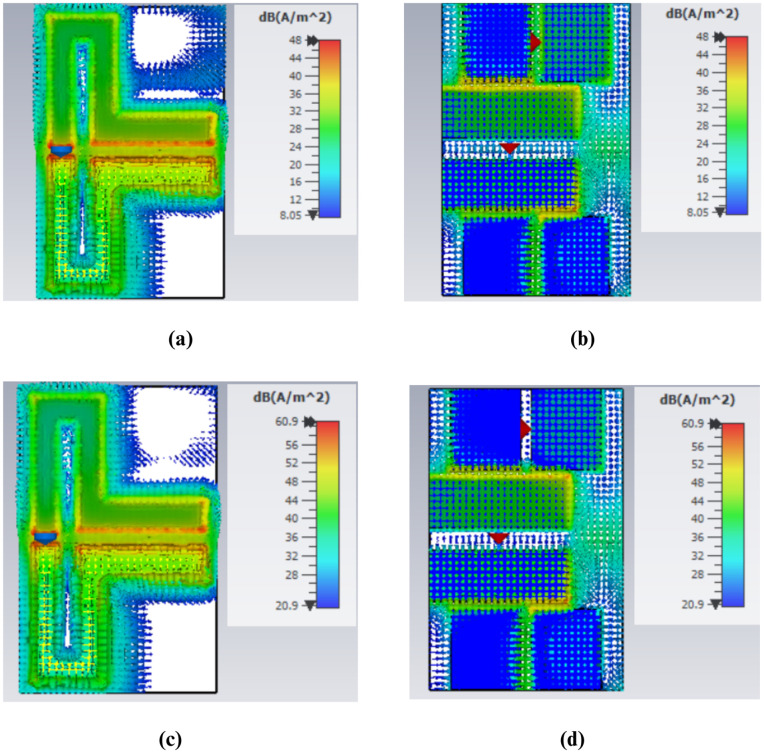
Surface Current Distribution at 0.43 GHz (a) Top View for Port 1 (b) Bottom View for Port ‌‌1 (c) Top View for Port 2 (d) Bottom View for Port 2.

The distribution of the surface currents in the front and back surfaces of the antenna at the frequency of 434 MHz is shown in Fig 7(a) to (d). The immense concentration of the current around length *L*_3_ implies that this particular part of the antenna is more sensitive to design changes, hence has a significant influence on the change in the reflection coefficient *S*_11_ as illustrated in Fig 2. Accordingly, any modification in this area may lead to an observable alteration in the performance of the antenna at the given frequency. Similarly, the high current on the backside at length, *L*_9_, contributes centrally to the *S*_11_ curve shift which is represented by Fig 9, making the use of the rear layer a critical factor in antenna tuning. Conversely, the lower current at length *L*_10_ indicates that this section of the antenna has a relatively small effect on the *S*_11_ curve, as is observed in Fig 5, indicating that the change at this length has a smaller effect on its total effect.

**Fig 8:**
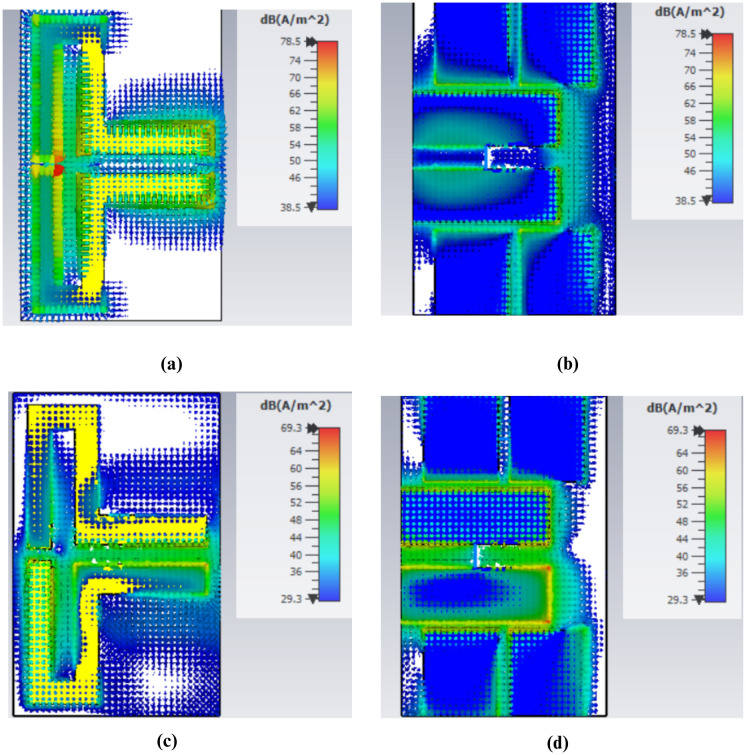
Surface Current Distribution at 5.7 GHz (a) Top View for Port 1 (b) Bottom View for Port 1 (c) Top View for Port 2 (d) Bottom View for Port 2.

The current distribution in the 5.7 GHz band is presented in Fig 8(a) – Fig 8(d). It is observable that the current density drops significantly at length *L*_10_ as indicated in Fig Fig 8(a) and Fig 8(c). This is equivalent to a small change of frequency as shown in Fig 5, which implies that this parameter does not have a strong influence on the general frequency behavior. However, current distribution indicates that the density of current on length *L*_9_ is greater, and it has a major impact on frequency shifting as observed in Fig 4.

**Fig 9:**
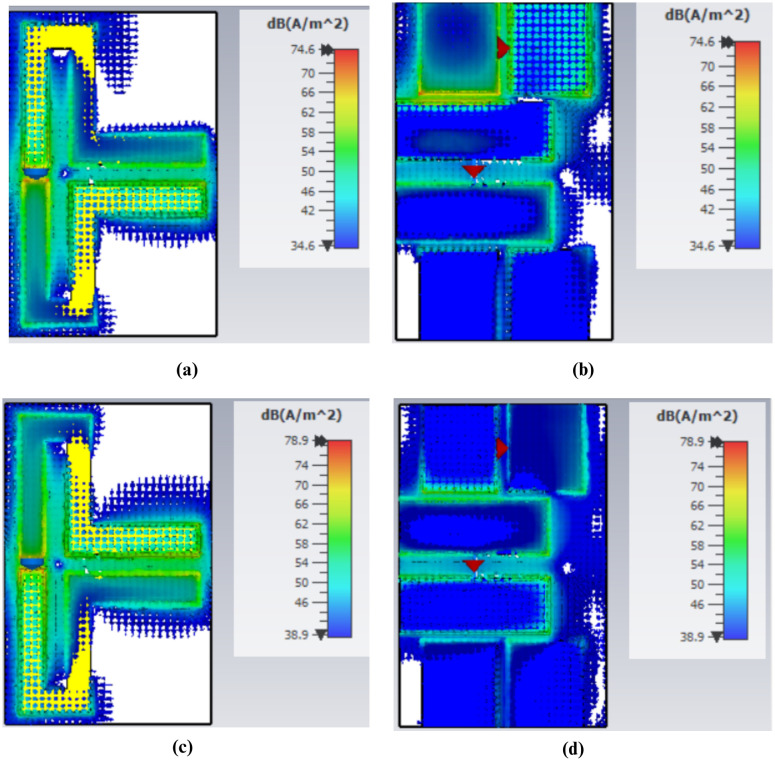
Surface Current Distribution at 4.8 GHz (a) Top View for Port 1 (b) Bottom View for Port 1 (c) Top View for Port 2 (d) Bottom View for Port 2.

For the 4.8 GHz band, the current distribution around length *L*_10_ is also quite low, indicating a minimal effect on the antenna’s performance, as shown in Fig 9. However, a stronger current is observed around length *L*_3_, which results in more noticeable variations in the antenna’s behavior, as reflected in Fig 2.

### Radiation patterns

The measured gain of this antenna is taken at resonant frequencies. Ports 1 and 2 had gains of −42 dBi and −29 dBi, respectively, at 434 MHz. The gains of the ports (port1 and port2) were −9 dBi and −5 dBi when the frequency shifted to 4.8 GHz. The gains at 5.7 GHz were 1.02 dBi and −6 dBi and the angular widths were 224° and 94°. Radiation efficiencies at 4.8 GHz and 5.7 GHz were −3.7 dB and −2.2 dB, respectively.

**Fig 10:**
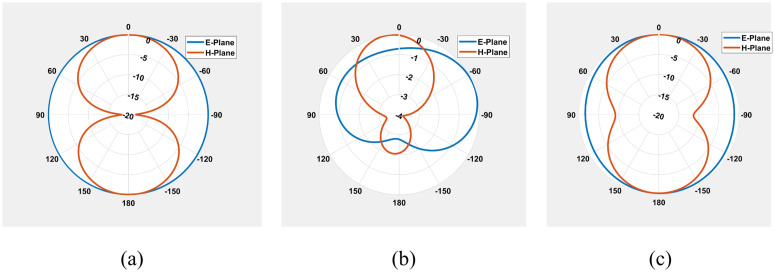
2D radiation gain patterns of the antenna at (a) 434 MHz ($\phi =0^{\circ },90^{\circ }$), (b) 4.8 GHz ($\phi =0^{\circ },90^{\circ }$), and (c) 5.7 GHz ($\phi =0^{\circ },90^{\circ }$).

Normalized radiation patterns for the mentioned frequencies are illustrated in Fig 10a to Fig 10c, illustrating consistent main‑lobe directions in both E‑ and H‑planes, with moderate variations in beam shape due to frequency scaling. These results indicates the antenna’s multi-band radiation stability over different angular directions.

### Testing antenna with cancer analyte

The properties of cancerous tissue analytes, especially relative permittivity, are dependent on a great number of factors in the case of electromagnetic simulation. These include type of cancer, pathological stage and field of frequency that is being studied. Due to differences in the electrical properties of various tissues, the same value cannot be applied to all types of cancers. The different circumstances require specific data to get more accurate results.

Researchers often rely on the data that is found in available literature in the form of the extant studies or databases informing about the electromagnetic properties of the tissues at particular frequencies. Since these properties are frequency-dependent, there is a requirement to ensure that values are chosen with reference to the operating range of the simulation.

As an example, in the applications that make use of microwave frequencies (common in cancer diagnostics) the relative permittivity of biological tissues (including malignant tissues) will generally be between 40 and 80. The electrical properties of malignant cells are necessary in simulating scenarios to identify them in comparison with healthy tissue cutaneous carcinoma.

In this regard, an antenna model was redesigned to include a cancer analyte in order to determine its effectiveness in the identification of a malignant cell. In the case of cutaneous carcinoma, cutaneous carcinoma was represented by using the following: $\epsilon_{r}$= 60, loss tangent = 0.001 *S*/*m* and density = 1000 *kg*/*m*^3^.

**Fig 11:**
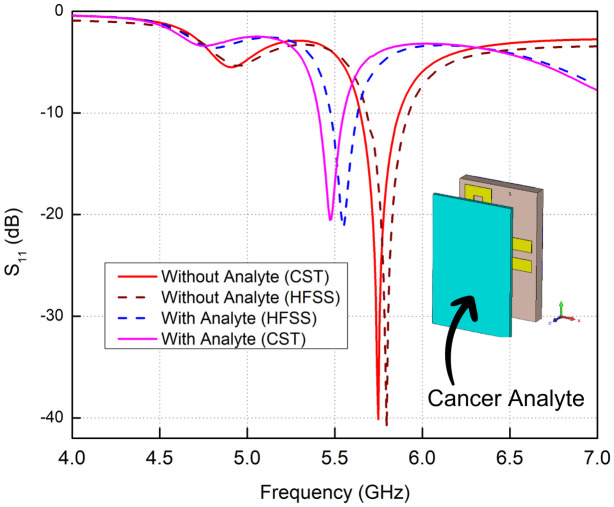
*S*_11_ results with Cancer Analyte at 5.7 GHz band using CST and HFSS.

The introduction of this analyte resulted in a frequency shift, moving from 5.75 GHz to 5.62 GHz, indicating the presence of cancerous cells. Even this slight shift could play an essential role in future cancer detection technologies. The results from CST Studio were compared with HFSS, as shown in Fig 11. Both simulations showed only slight differences in the return loss curve, with and without the cancer analyte, confirming the accuracy of the results.

The 5.7–5.8 GHz range in the ISM band has proven useful for biomedical applications, as shown by this simulation. The ability to detect even slight changes in frequency when cancerous tissue is present opens new possibilities for non-invasive cancer detection methods.

**Fig 12:**
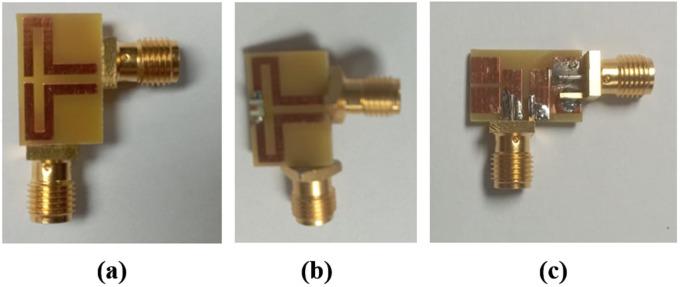
Fabricated prototype of the proposed shared aperture antenna.

Fig 12 shows the fabricated antenna. Fig 12(a) and (b) show the front view of the antenna without and with an inductor (lumped element), respectively. while Fig 12(c) illustrates the backside of the antenna.

**Fig 13:**
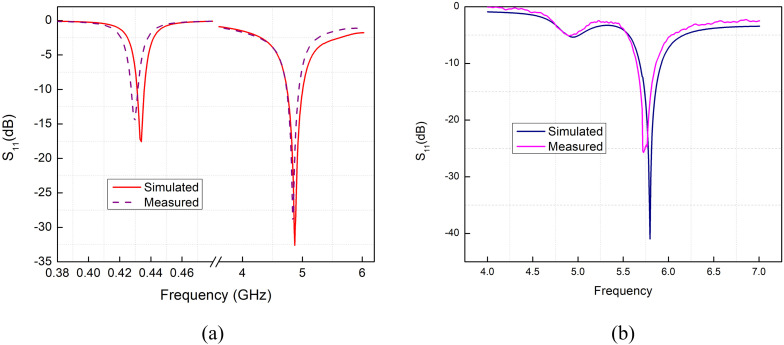
Comparison between simulation (CST Microwave Studio) and hardware measurement results. (a) 434 MHz and 4.8 GHz (b) 5.7 GHz.

Fig 13(a) and (b) shows a comparison between the simulated and measured *S*_11_ responses of this antenna design. The CST simulation curve and the hardware measurement follow the same overall trend, with both showing a strong resonance near 5.7 GHz. Although the measured result is slightly shifted and not as deep as the simulated one, the two curves are closely aligned, indicating that the fabricated prototype performs closely to the simulated design. This agreement confirms that the antenna behaves as expected when implemented in hardware.

In practice, the SMT inductor placed in the folded–dipole path does not behave exactly like the ideal lumped element used in the CST model. Real inductors have a specific self-resonant frequency (SRF), and their behavior begins to change as the operating frequency approaches this point. When the antenna operates near to the SRF of the inductor then it does not acts like a pure inductor, therefore the losses starts to appear.

The effective reactance of the inductor at 5.7 GHz in the presence of these frequency-dependent effects is not as predicted by the simulation. The difference in impedance between two points of the dipole disturbs the desired transformation of impedance in the dipole and may cause the resonance to shift slightly and reduce the depth of the return loss. The resulting curve of *S*_11_ will have a lower sharpness and can have a small frequency offset as compared to the CST prediction.

### Applications

The potential of the 434 MHz band in biomedical applications is significant, especially for wireless health monitoring and experimental studies. Remote patient monitoring and biotelemetry systems worn on animals under scientific investigation are an interesting potential use of this frequency band. The 434 MHz band is not traditionally assigned for medical use but can be considered suitable for some biomedical applications as long as the issues of the interference are alleviated and the regulations of the area are adhered to.

The 4.8 GHz antenna systems have diverse applications that are effective in various high-frequency applications such as the radar systems to precisely identify the target, the satellite communications to provide the link of ground-stations, and the Unmanned Aerial Vehicles (UAVs) to provide the high-speed and safe transmission of data. These concepts emphasize the significance of 4.8 GHz antennas in the development of the radar infrastructure, satellite connections dependability, and UAV communication infrastructure.

### Comparison with previous work

It is evident to the best of authors knowledge that hardly at the frequencies band mentioned, any shared aperture antenna is designed. The gains relative to the size of the antennas mentioned in the Table 2 can be compared with our proposed design. Additionally, the novel design presented in this paper uses a shared aperture for biomedical and communication applications. This multifunctionality is not evident in the comparison work mentioned in the Table 2. Also, the ability to support both communication and cancer-related spectral detection demonstrates the practical versatility and system-level value of the proposed antenna.

**Table 2:** Comparison of Shared/Co-Aperture Antenna Designs (2024–2025) Emphasizing Application, Size, and Gain.

Work	Frequency Bands	Size (mm / λ)	Gain (dBi)	Substrate	Application
**This Work (2026)**	0.431–0.435 GHz; 4.75–4.96 GHz; 5.64–5.88 GHz	**Size:** 16.40 × 10.50 × 1.52 mm In λ: 0.433 GHz: 0.0386λ × 0.0247λ × 0.0036λ 4.85 GHz: 0.431λ × 0.276λ × 0.040λ 5.76 GHz: 0.512λ × 0.328λ × 0.048λ	−31, −3, −6.8	FR-4	Communication + Cancer detection
[28]	2.45 GHz	**Size:** 17.28 × 14.28 × 0.28 mm In λ: 2.45 GHz: 0.141λ × 0.117λ × 0.0023λ	−32	Rogers TMM4	Implantable antenna
[29]	1.4 GHz; 2.45 GHz	**Size:** 6 × 6 × 0.254 mm In λ: 1.4 GHz: 0.028λ × 0.028λ × 0.0012λ 2.45 GHz: 0.049λ × 0.049λ × 0.0021λ	−32.7, −25.92	Rogers RT/Duroid 6010	Compact dual-band antenna for cardiac pacemaker
[30]	0.402 GHz	**Size:** 22.5 × 30 × 0.05 mm In λ: 0.402 GHz: 0.030λ × 0.040λ × 0.00007λ	−32	Polyamide	Implantable antenna (MICS-band)

### Advantages and limitations of proposed antenna

#### Advantages.

- The antenna is also very small in size, and it has been reduced to about 80 percent in size without losing its ability to work at various frequencies.
- Its shared-aperature design allows the simultaneous use of multiple bands in the same structure, making it especially attractive to biomedical equipment that is space-constrained.
- A unique property is the sensitivity to small frequency deviation near 5.7GHz, which has potential to indicate the presence of malignant cells, hence a potential future non-invasive modality of early cancer detection.

#### Limitations.

- Using an FR-4 substrate, the dielectric losses are increased, and so, a slight efficiency loss may be achieved compared to state-of-the-art low-loss dielectric materials.
- The acquisition of a suitable inductor with the specific specification necessary to achieve a miniaturization of the components is a mighty challenge by itself since this has a direct effect on the impedance matching and the performance of the antenna as a whole.
- Besides, the miniaturization process hinges on the integration of lumped components thus increasing the level of design complexity and could swell the manufacturing variables.
- The performance is expected to vary with varying tissue properties, and the calibration of the performance to provide accurate ‌‌oncological detection with a diverse population of patients.

## Conclusion

In this paper, we present a compact miniaturized antenna working in the multi-band sub-1 GHz and sub-6 GHz frequency ranges. It stands out from the rest by using a shared aperture, enabling multiple frequency bands to operate on a single small antenna. The antenna achieves sub-1 GHz resonance with an inductor in the folded dipole patch, enabling excellent impedance matching for biomedical applications. It is constructed on an FR-4 substrate of dimensions (16.40 × 10.50 × 1.52 *mm*^3^) (0.0386λ×0.0247λ×0.0036λ, 0.431λ×0.276λ×0.040λ, 0.512λ×0.328λ×0.048λ with ($\epsilon_{r}$ = 4.3), where the top layer features a folded dipole while coaxial feeds are present at the bottom side. This allows the antenna to cover the 0.431-0.435 GHz, 4.75-4.96 GHz, and 5.64-5.88 GHz bands. One notable aspect of this design is its potential to detect cancer as it can sense small resonant frequency shifts around 5.7 GHz through variations in the *S*_11_ response. These shifts may indicate changes in tissue properties‌‌ associated with cancer, enabling early-stage detection.
